# A Muscle-Invasive Bladder Cancer Patient With High Tumor Mutational Burden and RB1 Mutation Achieved Bladder Preservation Following Chemotherapy Combined With Immunotherapy: A Case Report

**DOI:** 10.3389/fimmu.2021.684879

**Published:** 2021-06-10

**Authors:** Chuanzhen Cao, Zhichao Fu, Yueping Liu, Aiping Zhou, Jianfei Wang, Jianzhong Shou

**Affiliations:** ^1^ Department of Urology, National Cancer Center/National Clinical Research Center for Cancer/Cancer Hospital, Chinese Academy of Medical Sciences and Peking Union Medical College, Beijing, China; ^2^ Research Institute, GloriousMed Clinical Laboratory (Shanghai) Co., Ltd., Shanghai, China; ^3^ Department of Radiology, National Cancer Center/National Clinical Research Center for Cancer/Cancer Hospital, Chinese Academy of Medical Sciences and Peking Union Medical College, Beijing, China; ^4^ Department of Medical Oncology, National Cancer Center/National Clinical Research Center for Cancer/Cancer Hospital, Chinese Academy of Medical Sciences and Peking Union Medical College, Beijing, China

**Keywords:** muscle-invasive bladder cancer, immunotherapy, chemotherapy, bladder-sparing, next-generation sequencing

## Abstract

Neoadjuvant chemotherapy followed by radical cystectomy is the standard of care for patients diagnosed with muscle-invasive bladder cancer (MIBC). However, urinary diversion following radical cystectomy significantly reduces patient quality of life. In addition, patients who significantly respond to neoadjuvant chemotherapy have a strong will to preserve the bladder. Bladder-sparing therapy has become a research focus worldwide. Although the bladder-sparing regimen, referred to as trimodality therapy (TMT), has been accepted, the efficacy of immunotherapy combined with chemotherapy for bladder preservation in patients with MIBC has not yet been published. We describe the case of a 50-year-old male presented intermittent macrohematuria and was diagnosed with bladder urothelial carcinoma by diagnostic transurethral resection of bladder tumor (TURBt) with clinical stage IIIA (cT3bN0M0). A complete response was achieved after four courses of neoadjuvant chemotherapy combined with pembrolizumab. Then, we performed a second TURBt plus randomized biopsy by cystoscopy. The pathology indicated no tumor in the bladder. Adjuvant chemoradiotherapy and immunotherapy were subsequently performed. Imaging examinations, cystoscopy and urine tumor DNA (utDNA) levels were used for surveillance after treatment. Finally, the patient achieved bladder preservation and had remained cancer-free for 19 months at the last follow-up on February 20, 2021. This is the first published case study to describe neoadjuvant chemotherapy plus pembrolizumab followed by concurrent chemoradiotherapy as a novel bladder-sparing regimen and successfully achieved a promising outcome.

## Introduction

Bladder cancer is the ninth most common cancer worldwide (1). Approximately 25% of patients with urothelial carcinoma were diagnosed with muscle-invasive bladder cancer (MIBC), which was an aggressive type (2–4). The recommended standard of treatment is neoadjuvant cisplatin-based chemotherapy followed by radical cystectomy (RC). Up to 50% of patients are ineligible to receive RC as a result of pre-existing contraindications (5). Due to the reduced quality of life after RC, some patients have a strong will to preserve their native bladders. How to achieve bladder preservation without influencing prognosis has become a research focus. Unfortunately, effective bladder-sparing options are limited. Immunotherapy, such as programmed cell death 1 (PD-1)/programmed death-ligand 1 (PD-L1) inhibitor treatment, has emerged as a prospective therapeutic approach for multiple solid tumors (6). In the PURE-01 phase II study, MIBC patients with clinical T2-3bN0M0 disease received pembrolizumab before RC, and 42% achieved a pathologic complete response (pCR) (7). This study indicated that pembrolizumab as neoadjuvant therapy could be a worthwhile regimen.

Herein, we report an innovative bladder-sparing regimen consisting of pembrolizumab and neoadjuvant chemotherapy followed by concurrent chemoradiotherapy.

Written informed consent to participate in this study was provided by the participant. Written informed consent was obtained from the individual for the publication of any potentially identifiable images or data included in this article.

## Case Presentation

**Figure 1:**
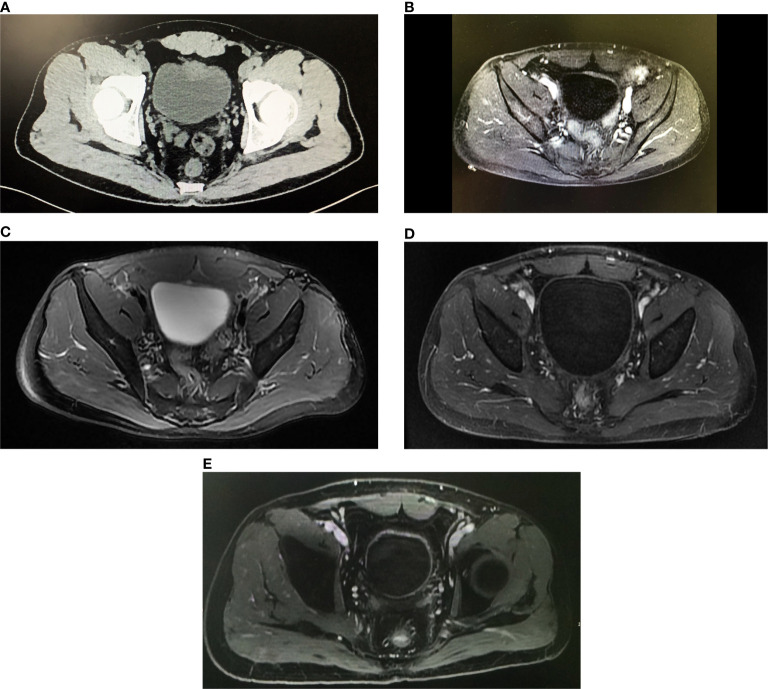
Imaging of the patient over the course of therapy. **(A)** The axial pelvic CT image before treatment demonstrated a mass was about 3.0 cm arising from the anterior wall of the bladder and the perivesical fat was invaded. **(B)** After two cycles of neoadjuvant therapy, the axial image from dynamic contrast-enhanced pelvic MRI indicated the thickness of enhanced anterior wall of the bladder decreased. **(C)** After four cycles of neoadjuvant therapy, the axial T2W image from pelvic MRI showed the light thickening of the anterior wall was persisting, but no node or tumor was found. **(D)** After concurrent chemoradiotherapy plus pembrolizumab, the axial contrast-enhanced MRI on arterial phase showed the bladder was normal with no sign of tumor recurrence. **(E)** The axial contrast-enhanced MRI on arterial phase still showed no tumor in the bladder at the last follow-up on February 20, 2021.

In April 2019, a 50-year-old man with a 10-year smoking history and a family history of lung and colorectal cancer experienced intermittent macrohematuria. Pelvic computed tomography (CT) revealed an irregular mass measuring 3.0 cm × 2.1 cm located at the anterior wall of the bladder (**Figure 1A**). Diagnostic transurethral resection of bladder tumor (TURBt) indicated that the tumor was cauliflower-like with broad base. The pathology revealed that the tumor was MIBC. As the chest/abdominal/pelvic CT scans indicated that the perivesical fat was invaded, the patient was diagnosed with clinical stage III-A (cT3bN0M0) bladder cancer.

The tissue sample collected during TURBt had a purity of 65% and was submitted for next-generation sequencing (NGS) analysis using a 642-gene panel. The tumor mutation burden (TMB) was 19.10 Mutants/Mb and the microsatellite state was stable (MSS). Furthermore, inactivating mutation in the *RB1* gene was detected (**Table 1**). The immunohistochemistry showed that the combined positive score (CPS) of the PD-L1 expression level was <1, as determined by using a monoclonal mouse anti-human PD-L1 clone (22C3) antibody, and the frequency of infiltrating CD8+ T cells was 2%.

**Table 1:** Results of gene mutation analysis of the patient tumor tissue.

Gene	Position	Base alteration	Amino acid alteration	Mutation abundance
*RB1*	Exon 7	c.2368C>T	p.Q217X	41.20%
*FBXW7*	Exon 11	c.1698G>A	p.W566X	56.50%
*ARID1A*	Exon 7	c.2368C>T	p.Q790X	1.20%
*ABRAXAS1*	Exon 8	c.709_710insT	p.E237fs	10.60%

Considering the promising efficacy of anti-PD-1 immunotherapy for patients with advanced bladder cancer and the high pCR rate in MIBC-related research, the patient strongly requested neoadjuvant chemotherapy combined with immunotherapy.

Since May 14, 2019, the patient received the neoadjuvant treatment, which included four cycles of gemcitabine and cisplatin (GC) plus concurrent pembrolizumab. The regimen consisted of gemcitabine (1,000 mg/m^2^) on days 1 and 8, cisplatin (60 mg/m^2^) on day 2, and pembrolizumab (200 mg) on day 2. As a grade 3 adverse event of bone marrow suppression arose, the gemcitabine and cisplatin doses were decreased to 850 mg/m^2^ and 50 mg/m^2^ in the following cycles. After two cycles of neoadjuvant therapy, pelvic MRI showed that the thickness of the anterior wall of the bladder was lessened, and the thickest area was only 0.5 cm (**Figure 1B**). The patient was considered to have achieved a partial response.

On July 28, 2019, pelvic MRI was repeated after the fourth cycle of neoadjuvant therapy. The imaging results showed that light thickening of anterior wall was persisting, but no node or obvious tumor was shown (**Figure 1C**), and the result of urine cytology analysis was negative. Given these results, the patient strongly preferred bladder-sparing treatment.

On July 30, 2019, the patient received the second TURBt and randomized biopsies by cystoscopy. The pathological analysis showed inflammation and interstitial edema without any tumor in the bladder and the stage was downgraded to T0.

Concurrent chemoradiotherapy started 1 month after the second TURBt. The regimen consisted of image-guided intensity-modulated conformal radiotherapy to the true pelvis with 45 Gy in 25 fractions plus the local lesion with 20 Gy in 10 fractions and concurrent cisplatin (40 mg/m^2^, once per week for 5 cycles). The side effects during chemoradiotherapy included second-degree fatigue and leukopenia. However, the patient recovered after symptomatic treatments. To evaluate the effect of chemoradiotherapy, the patient received pelvic MRI plus chest/abdominal CT on January 7 (**Figure 1D**) and cystoscopy on January 10, 2020. The results revealed that the bladder was normal with no sign of tumor recurrence. During this period, the patient received concurrent pembrolizumab (200 mg per 21 days). Finally, the patient achieved bladder preservation.

**Figure 2:**
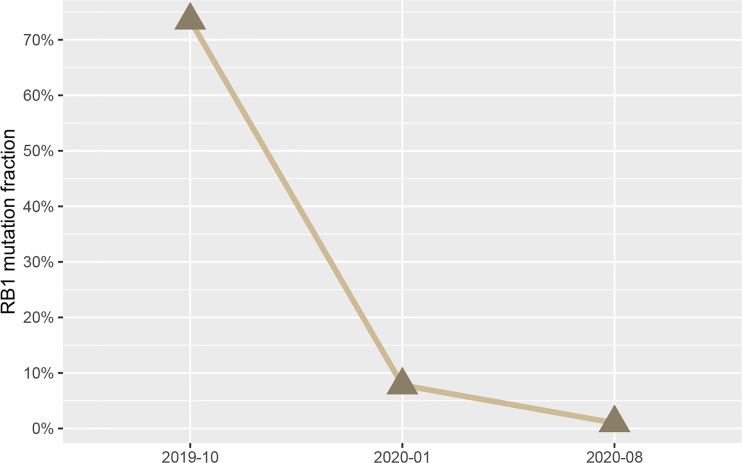
The mutation frequency of the *RB1* gene in the urine of the patient.

**Figure 3:**
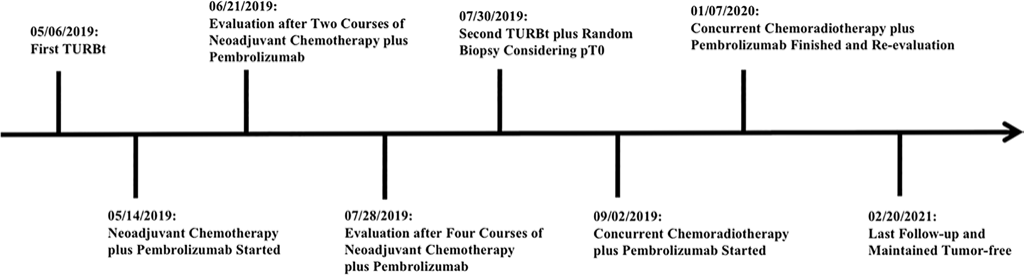
Timeline of patient treatment.

In addition, to monitor the status of the disease, urine tumor DNA sequencing analysis was carried out three times by a 642-gene panel from October 2019 to August 2020. The results showed that the mutation frequency of *RB1* decreased significantly (**Figure 2**). On February 20, 2021, MRI results showed that there was still no tumor in the bladder (**Figure 1E**). Cystoscopy and urine cytology analyses were also negative. The patient had maintained cancer-free status and excellent bladder function for 19 months at the end of follow-up. The overall treatment timeline is shown in **Figure 3**.

## Discussion

The latest National Comprehensive Cancer Network guidelines recommend that the standard of care for the treatment of MIBC is neoadjuvant cisplatin-based therapy combined with chemotherapy and subsequent radical cystectomy. However, some patients cannot accept urinary diversion surgery and instead seek a bladder-sparing treatment strategy as an alternative to radical cystectomy. The widely accepted bladder-sparing regimen for MIBC patients is the tri-modality therapy (TMT). Giacalone et al. reported that 475 patients with cT2-T4a MIBC who underwent TMT had 66% and 59% disease-free survival rates at 5 and 10 years, respectively, and the risk of salvage cystectomy at 5 years was 29% (8). This demonstrated that TMT can be offered as an effective therapy for patients seeking bladder preservation. However, only 6% to 19% of MIBC met the conditions for TMT. Although TMT therapy was recommended as an alternative regimen for selected MIBC patients, patients with MIBC unsuitable for TMT still desire bladder-sparing regimens. This study revealed an optimistic bladder-sparing outcome by adopting neoadjuvant chemotherapy plus pembrolizumab and subsequent concurrent chemoradiotherapy. It indicated that the frequency of *RB1* mutation might play a promising role in monitoring tumor recurrence.

The tumor suppressor gene *RB1* is mutated in approximately 14% of urothelial carcinomas and is important for DNA repair (9). Defects in DNA repair-associated genes confer sensitivity to chemotherapy in bladder cancer cell lines and animal models (10, 11). The results of another study showed that patients with genomic alterations in the DNA repair-associated gene *RB1* had better overall survival (p = 0.007) after three cycles of cisplatin-based neoadjuvant chemotherapy for MIBC (12). According to the presence of an inactivating mutation of *RB1* in our patient, the optimistic response to cisplatin-based neoadjuvant chemotherapy was similar to previous studies.

Pembrolizumab as a PD-1 inhibitor was approved by the Food and Drug Administration for the treatment of adult and pediatric solid tumors on June 16, 2020. In this case, the expression of PD-L1 was relatively low (CPS<1, TPS<1%), but a high TMB (19.10 mutants/Mb) was detected. These results suggested that the patient would benefit from pembrolizumab therapy. In the PURE-01 study, pembrolizumab neoadjuvant therapy before RC in patients with MIBC resulted in an impressively high proportion (42%) of patients with pT0 (7). This result indicated that pembrolizumab could be a worthwhile neoadjuvant therapy when limited to patients with PD-L1 positive or high-TMB (≥15 mut/Mb) tumors.

Since tumor-derived DNA can be released into circulation and mutations in circulating free DNA (cfDNA) can be detected in various biological fluids, the detection of urine tumor DNA by a high-throughput sequencing method for disease surveillance in bladder cancer has been proposed. Monitoring the recurrence of bladder cancer by utDNA analysis has been explored in previous studies. Christensen et al. found that a high frequency of *FGFR3* and *PIK3CA* mutations in the urine was associated with the progression and metastasis of bladder cancer (13). In another study, Dudley et al. indicated that urine tumor DNA could monitor the recurrence of bladder cancer, including monitoring *RB1* mutation status (14). In this case, the *RB1* mutation frequency gradually decreased during subsequent follow-up. Meanwhile, the imaging results suggested that the patient remained free from recurrence, which was consistent with the *RB1* frequency decrease. These results showed that imaging examinations combined with urine molecular monitoring may be conducive for follow-up after bladder preservation therapy.

The patient received a novel regimen of neoadjuvant chemotherapy plus pembrolizumab and subsequent chemoradiotherapy that successfully preserved his bladder with no immunotherapy-related adverse events. The findings presented in this case study indicate that neoadjuvant chemotherapy plus immunotherapy with subsequent concurrent chemoradiotherapy may be a bladder-preserving option for MIBC patients, especially for those with a high TMB and *RB1* mutation score, and that the frequency of *RB1* mutation may play a promising role in monitoring tumor recurrence.

## Data Availability Statement

The raw data supporting the conclusions of this article will be made available by the authors, without undue reservation.

## Ethics Statement

This study was reviewed and approved by the Domain-Specific Review Board, Cancer Hospital Chinese Academy of Medical Sciences. The patients/participants provided their written informed consent to participate in this study. Written informed consent was obtained from the individual(s) for the publication of any potentially identifiable images or data included in this article.

## Author Contributions

CC and ZF were responsible for writing the draft of the manuscript. CC, AZ, and JW researched data, contributed to discussion, wrote the manuscript, and reviewed/edited the manuscript. YL evaluated images and contributed these images to the manuscript. AZ and JS were responsible for analysis of data, data interpretation, and revision. All authors contributed to the article and approved the submitted version.

## Conflict of Interest

Authors ZF and JW were employed by GloriousMed Clinical Laboratory (Shanghai) Co. Ltd. 

The remaining authors declare that the research was conducted in theabsence of any commercial or financial relationships that could be construed as apotential conflict of interest.
